# Sulforaphane Causes Cell Cycle Arrest and Apoptosis in Human Glioblastoma U87MG and U373MG Cell Lines under Hypoxic Conditions

**DOI:** 10.3390/ijms222011201

**Published:** 2021-10-18

**Authors:** Giulia Sita, Agnese Graziosi, Patrizia Hrelia, Fabiana Morroni

**Affiliations:** Department of Pharmacy and BioTechnology—FaBiT, University of Bologna—Alma Mater Studiorum, Via Irnerio 48, 40126 Bologna, Italy; giulia.sita2@unibo.it (G.S.); agnese.graziosi2@unibo.it (A.G.); fabiana.morroni@unibo.it (F.M.)

**Keywords:** sulforaphane, hypoxia, glioblastoma, apoptosis, U87MG, U373MG

## Abstract

Glioblastoma multiforme (GBM) is the most prevalent and aggressive primary brain tumor. The median survival rate from diagnosis ranges from 15 to 17 months because the tumor is resistant to most therapeutic strategies. GBM exhibits microvascular hyperplasia and pronounced necrosis triggered by hypoxia. Sulforaphane (SFN), an isothiocyanate derived from cruciferous vegetables, has already demonstrated the ability to inhibit cell proliferation, by provoking cell cycle arrest, and leading to apoptosis in many cell lines. In this study, we investigated the antineoplastic effects of SFN [20–80 µM for 48 h] in GBM cells under normoxic and hypoxic conditions. Cell viability assays, flow cytometry, and Western blot results revealed that SFN could induce apoptosis of GBM cells in a dose-dependent manner, under both conditions. In particular, SFN significantly induced caspase 3/7 activation and DNA fragmentation. Moreover, our results demonstrated that SFN suppressed GBM cells proliferation by arresting the cell cycle at the S-phase, also under hypoxic condition, and that these effects may be due in part to its ability to induce oxidative stress by reducing glutathione levels and to increase the phosphorylation of extracellular signal-regulated kinases (ERKs). Overall, we hypothesized that SFN treatment might serve as a potential therapeutic strategy, alone or in combination, against GBM.

## 1. Introduction

Glioblastoma multiforme (GBM) is the most invasive and deadly primary brain tumor. GBM has an annual incidence of 3–5 cases per 100,000 people, while the disease can occur both in children and adults, median age at diagnosis is 65 years [1]. It is approximately 1.6 times more common in males than females, the reason for which remains uncertain. The vast majority of patients with glioblastoma do not have any identifiable risk factors for tumor development [2]. The only established non-genetic risk is a history of exposure to ionizing radiation [3]. The overall survival period for GBM patients without postoperative treatment is 3–6 months. The introduction of radiotherapy increased the survival period to 9–12 months. Temozolomide has been added to radiotherapy, resulting in yet another increase in survival. Despite this progress and the enormous progress in diagnostics, the treatment protocol for GBM has remained essentially unchanged in past years [4]. For this reason, novel therapeutic strategies are urgently needed to treat this lethal disease. GBM is characterized by a heterogeneous microenvironment that considerably complicates mechanistic studies of GBM cell invasion. Furthermore, GBM undergoes malignant progression under hypoxic conditions [5]. Hypoxia is a feature found in several solid tumors, and it indicates a poor prognosis. Hypoxia drives malignancy by promoting chemo- and radiotherapy resistance, alters the tumor cells’ metabolism, generates strong genome instability, and increases angiogenesis [6]. Oxidative stress is critically involved in the development of malignant tumors [7]. Cancer cells can adapt to maintain redox homeostasis through a variety of mechanisms. Due to the poor vascularization of solid tumors and rapid nutrient consumption by tumor cells, oxygen and nutrients such as glucose in the tumor microenvironment are often limited [8]. Cancer cells develop metabolic adaptation to the tumor microenvironment, exhibiting metabolic flexibility (alternative material and energy sources) and metabolic plasticity (rewired metabolic pathways) [9]. These metabolic modifications not only increase key metabolic pathways such as glycolysis, pentose phosphate pathway (PPP), and glutaminolysis but also interact with multiple oncogenic signaling pathways such as phosphoinositide 3-kinase/protein kinase B- (PI3K/AKT), Ras-, p53-, Myc-, and reactive oxygen species (ROS)-related pathways [10]. Several studies have demonstrated massive production of ROS and the negative regulation of antioxidant system in cancer cells, because of hypoxia and metabolic alterations. In these conditions, different oncogenes are activated to stimulate cellular proliferation, transformation, and metastasis [11]. Mitochondria are central organelles at the crossroad of various energetic metabolisms. In addition to their pivotal roles in bioenergetic metabolism, they control redox homeostasis, biosynthesis of macromolecules and apoptotic signals, all of which are linked to carcinogenesis. Mitochondria also represent “stress sensors” that coordinate metabolic adaptation of cells to their microenvironment [12]. A decline in antioxidant capacity arising from genetic mutations may increase the mitochondrial flux of free radicals resulting in misfiring of cellular signaling pathways. Often, a metabolic reprogramming arising from these mutations in metabolic enzymes leads to the overproduction of so called ’oncometabolites’ in a state of ‘pseudohypoxia’. This can inactivate several of the intracellular molecules involved in epigenetic and redox regulations, thereby increasing oxidative stress and giving rise to growth advantages for cancerous cells [13]. Glutathione (GSH) is the most important antioxidative agent in cells and is essential in the detoxification of carcinogens [14]. It is reported that targeting GSH could be a potential strategy to render cancer cells more sensitive to the standard therapies [15].

Sulforaphane (SFN), 1-isothiocyanate-4-(methylsulfinyl)-butane is an isothiocyanate (ITC) from cruciferous vegetables, in particular broccoli and broccoli sprouts [16,17]. By monitoring quinone reductase induction in cultured murine hepatoma cells in biological assays, Zhang et al. were able to isolate SFN from broccoli. Approximately 9 mg of SFN was isolated from 640 g of fresh broccoli florets (ca. 14 μg/g of fresh weight) [18]. SFN has attracted the attention of researchers for its remarkable properties in disease treatment, and very low toxicity in healthy tissue [19]. Indeed, it shows pleiotropic activities by modulating several pathways involved in the pathogenesis of cancer [20]. The chemopreventive effects of the ITCs are traditionally attributed to their ability to prevent tumorigenesis through enhancement of carcinogen phase 2 detoxification, which is strictly related to the translocation of the nuclear factor NF-E2–related factor 2 (Nrf2) into the nucleus [21]. Nrf2 controls both the basal and stress-inducible expression and function of key metabolic components belonging to metabolic reactions, such as GSH biosynthesis and recycling [22]. Moreover, SFN can easily cross the blood-brain barrier (BBB) and reach the central nervous system (CNS) after intraperitoneal administration [23]. Recent studies have demonstrated the possible application of SFN in GBM by promoting apoptosis and inhibiting both growth and invasion of tumoral cells. Moreover, SFN has demonstrated the feasibility of targeting the chemoresistance of cancer cells [24].

Despite the recognized properties of SFN in relation to cancers, little information is available on the properties of SFN in GBM under hypoxic condition, which is a distinct feature of this tumor. Here, we investigated the effects of SFN on U87MG and U373MG GBM cells. Our study is aimed at providing a new insight for a novel potential therapeutic agent in the treatment of GBM. Our results showed that SFN causes cell-cycle arrest and reduces cell growth in GBM cells also under hypoxic condition and these effects may be attributed to its ability to increase oxidative status and phosphorylation of extracellular signal-regulated kinases (ERKs).

## 2. Results

### 2.1. The Effects of SFN on Cell Viability

**Figure 1:**
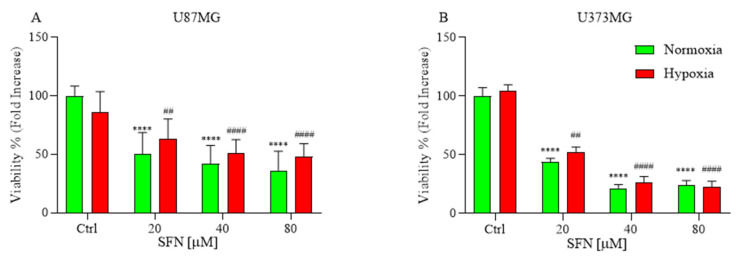
Effects of SFN on cell viability after 48 h of treatment [20–80 µM] in normoxic (green) or hypoxic (red) conditions. All SFN concentrations induced a significant decrease in cells viability in U87MG (**A**) and U373MG (**B**) cell lines. Data are expressed as fold increases in the percentage of cell viability versus corresponding controls and reported as mean ± SD of three independent experiments (**** *p* < 0.0001 vs. normoxic Ctrl group; ^##^ *p* < 0.01 and ^####^ *p* < 0.0001 vs. hypoxic Ctrl group. Two-way ANOVA, post hoc test Bonferroni).

The anti-proliferative effect of SFN was estimated using alamarBlue assay under hypoxic and normoxic conditions (Figure 1). Hypoxia is crucially involved in tumor aggressiveness by causing resistance to radiotherapy and chemotherapy [25]. Nevertheless, we did not observe any cell culture suffering under severely hypoxic conditions. The cells were incubated with various concentration of SFN [20–80 µM] under hypoxic or normoxic conditions for 48 h. The results indicated that all SFN concentrations induced a significant decrease in cells viability in both U87MG (Figure 1A) and U373MG (Figure 1B) cell lines. Interestingly, in all tested cell lines, SFN decreased cell viability in a comparable manner in both normoxia and hypoxia.

### 2.2. The Effects of SFN on Apoptotic Cell Death

We hypothesized that the reduced cell number observed upon SFN treatment might be associated with apoptosis. SFN has already demonstrated apoptotic effects on different GBM cells in normoxia [26,27,28]. Therefore, SFN-treated cells were assessed for the classical apoptosis indicators, caspases activation, and DNA fragmentation.

**Figure 2:**
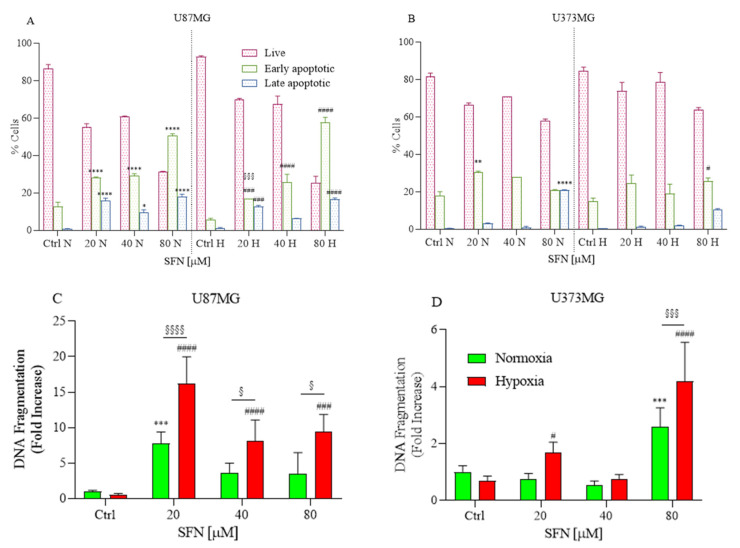
Effects of SFN on caspases-3/-7 (**A**,**B**) and DNA fragmentation (**C**,**D**) after 48 h of treatment [20–80 µM] in normoxic or hypoxic conditions. Caspases-3/-7 were detected in U87MG (**A**) and U373MG (**B**) cell lines to identify early (green) and late (blue) apoptosis. The SFN treatment induced apoptosis at 48 h of treatment both in normoxic and hypoxic conditions. DNA fragmentation was detected in U87MG (**C**) and U373MG (**D**) and SFN induced apoptosis in normoxic (green) and hypoxic (red) conditions. Data are expressed as percentage of live, early and late apoptotic cells (**A**,**B**) or as fold increases in DNA fragmentation versus corresponding controls (**C**,**D**) and reported as mean ± SD of three independent experiments (* *p* < 0.05, ** *p* < 0.01, *** *p* < 0.001 and **** *p* < 0.0001 vs. normoxic Ctrl group; ^#^ *p* < 0.05, ^###^ *p* < 0.001, and ^####^ *p* < 0.0001 vs. hypoxic Ctrl group; ^§^ *p* < 0.05, ^§§§^ *p* < 0.001 and ^§§§§^ *p* < 0.0001 vs. the respective normoxic treatment group. Two-way ANOVA, post hoc test Bonferroni).

Caspases are key proteases of apoptosis; in order to examine whether caspase activity was related to apoptosis in U87MG and U373MG cells, a multi-caspase assay was performed using the Muse™ Cell Analyzer after cells were exposed to different concentrations of SFN (Figure 2A,B) under hypoxic and normoxic conditions.

After the treatment, the cells that were positive for caspase-3/-7 and 7-AAD staining were detected. The ratio of apoptotic cell populations, early and late apoptosis, increased compared to the higher concentration of SFN. Thus, in both tested cell lines and normoxic and hypoxic conditions, 48 h of SFN treatment significantly induced early- and/or late-stage apoptosis, and the highest proportion of apoptotic cells was found in the U87MG cell line.

Additionally, another key feature of apoptosis, DNA fragmentation, was also investigated using an ELISA kit. The results of DNA fragmentation assay consistently indicated that SFN induced apoptosis in both cell lines (Figure 2C,D). The apoptotic effect of SFN is more marked in hypoxia than normoxia, especially for U87MG, where it is already significant at the concentration of 20 µM (Figure 2C).

### 2.3. The Effects of SFN on Cell Cycle and Cell Motility

**Figure 3:**
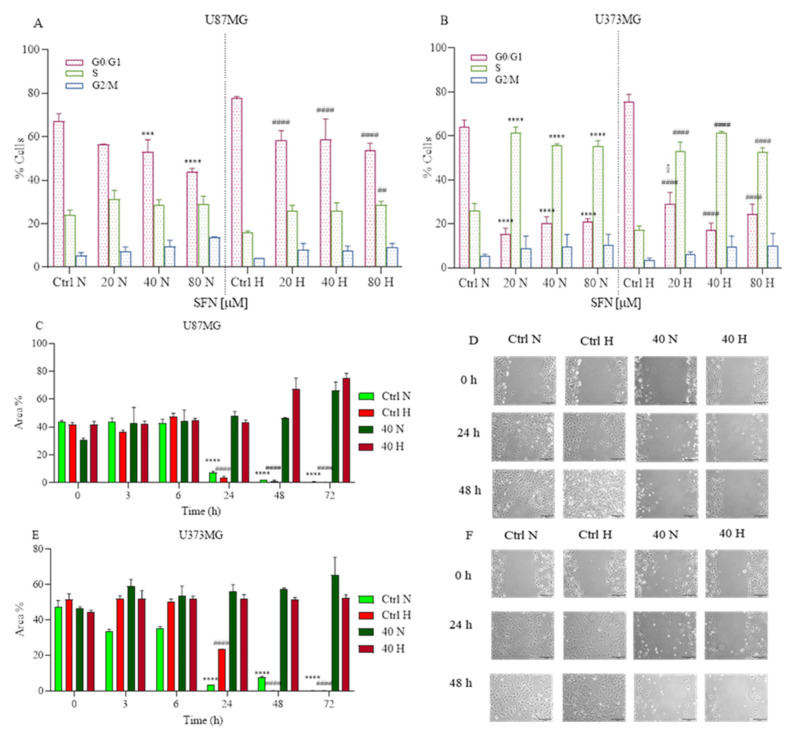
Effects of SFN on cell cycle (**A**,**B**) after 48 h of treatment [20–80 µM] and cell motility after [40 µM] treatment up to 72 h in normoxic or hypoxic conditions. SFN reduces GBM cell proliferation increasing the number of cells in the S-phase in both U87MG (**A**) and U373MG (**B**) cell lines. Cell motility showed in U87MG (**C**,**D**) and U373MG (**E**,**F**) that SFN suppressed GBM cell migration and proliferation both in hypoxic and normoxic conditions. Quantitative analysis of the area percentage of the wound not covered by U87MG (**C**) and U373MG (**E**) cells. Representative images of U87MG (**D**) and U373MG (**F**) cells at 0 h, 24 h and 48 h, 10× magnification, scale bar 100 µm. Data are expressed as the percentage of cells in the G0/G1, S, and G2/M phases (**A**,**B**) or as the percentage of the wound area not covered (**C**,**E**) and reported as mean ± SD of three independent experiments (**A**,**B**): *** *p* < 0.001 and **** *p* < 0.0001 vs. normoxic Ctrl group; ^##^ *p* < 0.01 vs. hypoxic Ctrl group; ^§^ *p* < 0.05, vs. the respective normoxic treatment group. (**C**,**D**): **** *p* < 0.0001 vs. normoxic Ctrl group at time 0 h; ^####^ *p* < 0.0001 vs. hypoxic Ctrl group at time 0 h. Two-way ANOVA, post hoc test Bonferroni).

Prompted by the above findings, we examined whether SFN influenced the cell cycle of GBM cells. SFN has been implicated in the regulation of cell proliferation in human cancer cells in several research studies [19,29]. The GBM cells were treated with 20, 40 and 80 μΜ of SFN for 48 h under hypoxic or normoxic conditions. Flow cytometry analysis of cell cycle distribution revealed that as SFN concentration increased, the number of cells in the S-phase increased in U87MG (Figure 3A) and U373MG (Figure 3B) cells in both normoxic and hypoxic conditions.

These results indicated that SFN suppressed GBM cell proliferation by arresting the cell cycle at the S -hase and this effect was more significant in the U373MG cells than in the U87MG cells.

Subsequently, we examined whether SFN has any effect on GBM cell motility. The monolayer cells of U87MG and U373MG were wounded and treated with 40 µM of SFN up to 72 h in normoxic and hypoxic conditions.

Cell motility after wound generation revealed that untreated cells migrated more than SFN treated cells (Figure 3C–F). These results indicated that SFN suppressed GBM cell migration and proliferation both in hypoxic and normoxic conditions. Interestingly, SFN has already demonstrated invasion inhibiting effects in GBM cells by influencing E-cadherin, Galectin-3 and matrix metalloproteinases 2 and 9 [26].

### 2.4. The Effects of SFN on Mitochondrial and Oxidative Status

**Figure 4:**
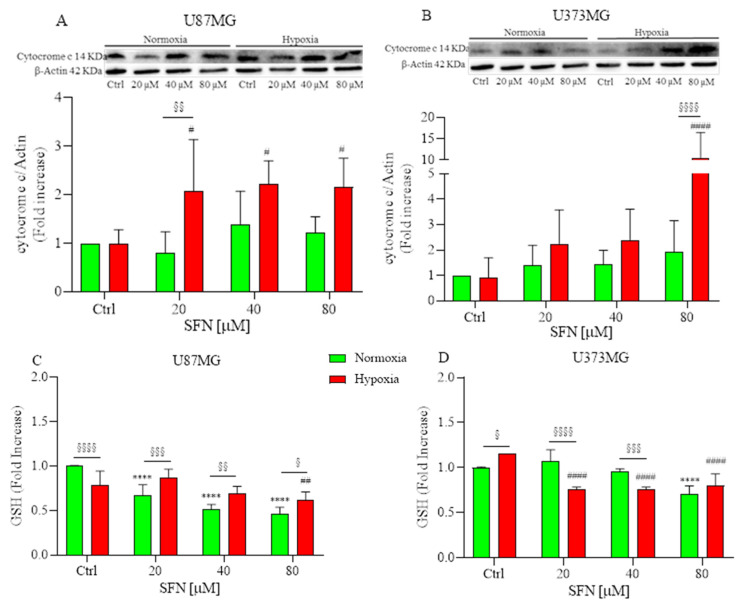
Effects of SFN on mitochondrial (**A**,**B**) and oxidative status (**C**,**D**) after 48 h of treatment [20–80 µM] in normoxic (green) or hypoxic (red) conditions. (**A**,**B**) top: representative images of cytochrome C and β-actin expressions in U87MG (**A**) and in U373MG (**B**) cells. (**A**,**B**) bottom: quantitative analysis of the Western Blotting results. SFN treatment significantly increased the release of cytochrome c under hypoxia in both cell lines. SFN treatment reduced the levels of GSH in a dose-dependent manner both in normoxic and hypoxic conditions (**C**,**D**). Data are expressed as fold increases and reported as mean ± SD of three independent experiments (**** *p* < 0.0001 vs. normoxic Ctrl group; ^#^ *p* < 0.05, ^##^ *p* < 0.01 and ^####^ *p* < 0.0001 vs. hypoxic Ctrl group; ^§^ *p* < 0.05, ^§§^ *p* < 0.01, ^§§§^ *p* < 0.001 and ^§§§§^ *p* < 0.0001 vs. the respective normoxic treatment group. Two-way ANOVA, post hoc test Bonferroni).

Two distinct pathways of apoptotic cell death have been described: the intrinsic mitochondrial-mediated pathway and the extrinsic death receptor (DR)-mediated pathway [30]. The collapse of mitochondrial structural integrity is an early event of the intrinsic apoptotic pathway [31]. In this pathway, a variety of apoptotic stimuli cause cytochrome c release from mitochondria, which in turn induces a series of biochemical reactions that result in caspase activation and consequent cell death [32]. Subsequently, the expression of cytochrome c protein in U87MG and U373MG cells was assayed by Western Blotting. As shown in Figure 4A,B, under hypoxia, SFN significantly increased the release of cytochrome c, and this effect is more significant in U87MG than in U373MG cells, suggesting that SFN could induce apoptosis through the mitochondria-dependent pathway. These results confirm Zhang’s study in which SFN induced apoptosis in GBM cells most likely through a Bad–Bax/Bcl-2-cytochrome C signaling pathway [26].

ROS-mediated oxidative damage represents one of the most important mechanisms of drug-induced cancer-cell apoptosis [33]. Therefore, the oxidative status of SFN-treated U87MG and U373MG cells was investigated by measuring GSH levels. Cells require the maintenance of cellular redox balance, and GSH represents the principal cellular redox buffer. Interestingly, SFN reduced cellular GSH levels in colon cancer cell lines comparable to that caused by phenethyl ITC (a compound previously reported to induce GSH depletion [34]) suggesting that SFN elevates ROS at least in part by disabling the GSH antioxidant system [35]. We hypothesized that the ROS excess was caused by a flaw in the antioxidant system, which is incapable of removing ROS. Consequently, we investigated whether SFN exacerbates oxidative stress by causing depletion of intracellular GSH. As shown in Figure 4C,D, treatment with SFN considerably reduced the levels of GSH in a dose-dependent manner both in normoxic and hypoxic conditions.

### 2.5. The Effect of SFN on MAPK Signaling Pathway

**Figure 5:**
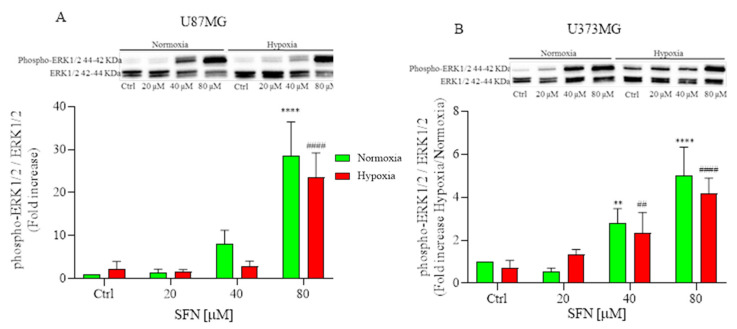
Effects of SFN on MAPK signaling pathway after 48 h of treatment [20–80 µM] in normoxic (green) or hypoxic (red) conditions. Top: representative images of phospho-ERK1/2 and total ERK1/2 expressions in U87MG (**A**) and in U373MG (**B**) cells. Bottom: quantitative analysis of the Western Blotting results. In U87MG cells (**A**) SFN treatment induced the phosphorylation of ERK at 80 µM, while in U373MG cells (**B**) the phosphorylation was dose-dependent both in hypoxic and normoxic conditions. Data are expressed as fold increases and reported as mean ± SD of three independent experiments. (** *p* < 0.01, **** *p* < 0.0001 vs. normoxic Ctrl group, ^##^ *p* < 0.01, ^####^ *p* < 0.0001 vs. hypoxic Ctrl group. Two-way ANOVA, post hoc test Bonferroni).

The mitogen-activated protein kinase (MAPK) signaling pathway has been linked to the pharmacological effects of some ITCs [36]; thus, we detected the effects of SFN on human GBM cells. As shown in Figure 5, SFN significantly activated ERK1/2 in human GBM cells after 48 h treatment, which was in agreement with previous studies that demonstrated SFN-cysteine, an analog of SFN, contributed to the phosphorylation of ERK1/2 in GBM cells [37]. However, the activation of ERK was considerably different between two human GBM cell lines. The phosphorylation of ERK was significant only at 80 µM in U87MG (Figure 5A); while the phosphorylation was dose-dependent in U373MG and significant at 40 µM both in hypoxic and normoxic conditions.

## 3. Discussion

Although surgery and radiation represent the first line of intervention in the early stages of GBM and very few approved drugs are available, GBM’s resistance to chemotherapy and radiation is a significant factor contributing to the aggressive clinical courses and to the poor prognosis [38]. The search for new chemo-preventive and/or chemo-therapeutic agents is a crucial and challenging approach to enhance the current state of unsatisfactory outcomes.

As well as in many other tumor types, the presence of hypoxic areas in GBM may contribute to chemo- and radio-resistance [39,40]. The uncontrolled proliferation of cancer cells, which outgrow their blood supply, deprives cells of nutrients and oxygen. Hypoxia is a pathophysiological condition that generally arises due to the fast proliferation of cancer cells as they grow beyond the blood supply, consequently depleting cells of nutrients and oxygen [41]. In many solid tumors, neovessels are often abnormal, immature, and leaky. Neovasculogenesis maintains blood flow to the growing tumor tissue that expands rapidly, providing nutrients and oxygen for thriving cancer cells; however, this condition means more demand causing even more hypoxia. Again, hypoxia in turn stimulates angiogenesis to ameliorate hypoxic condition, closing the vicious circle. As a consequence, the tumor tissue ends up being highly hypoxic with excessive but dysfunctional vasculature [42]. The abnormal and malfunctioning vessels play a critical role in generating necrotic and hypoxic regions, where residing cancer stem cells are protected from therapeutic agents, facilitating tumor aggressiveness as well as GBM stem cell proliferation [43,44,45]. Thus, new strategies to overcome resistance to treatment are needed in the care of GBM patients. Despite extensive effort in basic, translational, and clinical research, the treatment outcomes for patients with GBM are almost unchanged over the past 15 years. GBM is one of the most immunologically “cold” tumors, in which cytotoxic T-cell infiltration is minimal, and myeloid infiltration predominates. This is due to the profound immunosuppressive nature of GBM, a tumor microenvironment that is metabolically challenging for immune cells, and which contributes to the poor results obtained from immunotherapy [46].

Several epidemiologic studies have demonstrated that ITCs have an extensive range of different clinical applications and their carcinogenesis-inhibiting activities have been widely confirmed both in vitro and in vivo [47,48,49,50]. ITCs have also highlighted cytotoxic and antiproliferative effects in the micromolar range across several tumor cell lines, including neuroblastoma, breast cancer, prostate cancer, leukemia, bladder cancer, colorectal cancer and lung cancer in a dose- and time-dependent manner [51]. These findings point to the potential use of ITCs as bio-active compounds, justifying their possible clinical application, including in combination with other therapies. Notably, a growing interest has pointed to the possible effects of SFN in GBM not only to induce apoptosis, but also to inhibit growth and invasiveness of GBM cells [24,52,53]. Moreover, Bijangi et al. made an interesting finding that SFN is able to reduce the survival of GBM cells, GBM stem-cell-like spheroids, and tumor xenografts via multiple cell signaling pathways, while having no impact on the survival of healthy human brain cells in both in vitro and in vivo experiments [52]. Furthermore, Kumar et al. demonstrated that SFN could modulate the immune response of GBM cells. In particular, SFN inhibited the transformation of normal monocytes to myeloid-derived suppressor cells (MDSCs) by glioma-conditioned media in vitro at pharmacologically relevant concentrations that are non-toxic to normal leukocytes. MDSCs are a key component of the GBM immunosuppressive environment that allows it to evade immunosurveillance [54].

To the best of our knowledge, there are very few studies on the effects of SFN in the hypoxic tumor microenvironment and none specifically on GBM. Here, we have taken advantage of all the information about SFN and GBM to elucidate that SFN may be considered a valid therapeutic alternative in the hypoxic condition that characterizes GBM, as a result, the cell-cycle is arrested, and cell growth is inhibited in U87MG and U373MG cells. Although there are concerns about the provenance of the U87MG cell line, it is still assumed to be a glioma cell line [55].

In this study, SFN has the potential to significantly reduce cell viability and induce apoptosis at 20 µM in U373MG and U87MG cells. We also proved that SFN activated caspase-3 associated with the activation of ERK1/2 signaling pathway. The release of cytochrome c from the mitochondria may be the cause of caspase-3 activation, implying that SFN induced cell apoptosis in human GBM cells via the intrinsic apoptosis pathway. These data agree with the study by Karmakar et al. in which they found increase in cytosolic and decrease in mitochondrial cytochrome c levels in GBM cells following SFN treatments, strongly implying the participation of mitochondrial cytochrome c release in apoptosis mediation [27]. Even more interesting, in our study, these effects are maintained, even improved, under hypoxic condition, which may indicate how SFN can be effective even in the characteristic microenvironment of GBM.

In cancer cells, cyclic abnormalities and anti-apoptosis effects are common, and the capacity to induce cell cycle arrest and enhance apoptosis is a consideration in choosing potential chemotherapeutic agents [56,57]. The results of our study showed that SFN induced S-phase arrest and decreased invasion in U87MG and U373MG cells, which was consistent with previous reports by Wang et al. that SFN provokes S-phase arrest via p53-dependent antiproliferation and apoptosis induction in gastric cancer cells [19]. One of the most common abnormalities in gliomas is mutations in the p53 (also known as TP53) gene. The two cell lines used in the study have substantially different effects on the status of p53, the U373MG expresses mutant p53 protein, while the U87MG expresses wild-type p53 protein [58]. The wild-type protein corresponds to a functioning protein, which should be able to induce apoptosis, while the mutated protein is associated with an inactive form of p53, which fails to trigger apoptotic cell death. The U87MG probably eludes apoptosis through the overexpression of p53 inhibitors, such as Murine Double Minute-2 (MDM2) or p21, while in the mutated form (i.e., U373MG) the evasion of apoptosis is linked to the inactivation of p53 [58]. Interestingly, our results indicated that the effect of SFN was slightly different in the two cell lines. In particular, the anti-apoptotic effect, demonstrated by induction of caspase-3/-7 and DNA fragmentation and by the release of cytochrome c, was more conspicuous and dose-dependent in U87MG than in U373MG, probably because SFN could induce apoptosis in human GBM cells via the p53-dependent mitochondrial pathway, though the precise molecular mechanism remains unknown.

However, it should be noted that SFN is a pleiotropic compound and showed an antiapoptotic effect, even on U373MG, modulating some important pathways independently of the cellular p53 status. Many researchers demonstrated that p53 mutant isoforms increase overall ROS levels in cancer cells via an organized control of multiple redox-related enzymes and signaling pathways, thereby promoting cancer cell proliferation [59]. U373MG cells express the mutant p53 protein, so we can hypothesize that they could be particularly responsive to a pro-oxidant environment. Therefore, SFN stimulated cell death by causing redox imbalance, as demonstrated by the decrease in GSH levels and the increase in ERK1/2 phosphorylation.

The main kinase in the MAPK pathway, ERK1/2, is triggered via phosphorylation and repressed by its specific phosphatases. The MAPK signaling cascade transfers extracellular stimuli into cells and regulates many key elements of cell physiology. Transient phosphorylation of ERK1/2 (5–15-min stimulation) promotes cell growth [60], while sustained phosphorylation of ERK1/2 (>15 min stimulation) is responsible for cell apoptosis [61]. In recent studies, SFN significantly increased the phosphorylation of ERK1/2 in different cancer cell lines [62], which is closely related to cell division, invasion, and apoptosis [63,64]. Accordingly, in human GBM U87MG and U373MG cells, SFN inhibited invasion by activating ERK1/2 signaling. Therefore, we hypothesized that SFN could induce apoptosis in both hypoxic and normoxic conditions by activating ERK1/2.

In conclusion, we thought that SFN might activate ERK1/2, decreasing GSH levels and upregulating cleaved caspase 3, which resulted in cell apoptosis and decreased cell motility in U373MG and U87MG cells both in hypoxic and normoxic conditions.

## 4. Materials and Methods

### 4.1. Cell Culture and Treatments

Human GBM cell lines U87MG and U373MG were purchased from the Lombardy and Emilia Romagna experimental Zootechnic Institute (Italy). Cells were grown at 37 °C in a humidified incubator with 5% carbon dioxide (CO_2_) in Dulbecco’s modified Eagle Medium with phenol red (DMEM, Euroclone Spa, Pero, Milan, Italy) supplemented with 10% fetal bovine serum (FBS, Euroclone), 2 mM L-glutamine (Sigma-Aldrich, St. Louis, MO, USA), 50 U/mL penicillin and 50 μg/mL streptomycin (Sigma-Aldrich). For the hypoxic exposure, cells were placed in a sealed, self-contained Hypoxia Incubator Chamber (StemCell Technologies Inc., Vancouver, BC, Canada) connected to a Single Flow Meter (StemCell Technologies Inc.) for the precise control of gas flow to generate a hypoxic environment. After 10 minutes of insufflation a severe hypoxia environment was created, and the cells were kept in hypoxia as long as the SFN treatment (48 h). For the treatment with SFN (LKT Laboratories, St. Paul, MN, USA), a stock solution was prepared in dimethyl sulfoxide (DMSO) at 10 mM, then further diluted in complete medium to obtain a concentration range of 20–80 µM. The concentrations used were chosen on the basis of previous in vitro studies on GBM [26,52]. In each experiment cells were treated with different concentrations of SFN [20–40–80 µM] in DMEM 5% FBS under hypoxic (0.1% O_2_, 5% CO_2_, and 94.9% N_2_) or normoxic (5% CO_2_) conditions.

### 4.2. Determination of Cell Viability

The cell viability was evaluated by the alamarBlue HS™ Cell Viability Reagent (Invitrogen Corporation, Waltham, MA, USA) as an indicator of the cell health through the reducing power of living cells. Once inside the live cell, resazurin is reduced to resorufin, a red and highly fluorescent dye [65]. Briefly, U87MG and U373MG cells were seeded in a 96 well plate at 1 × 10^4^ cells/well, incubated for 24 h and treated with SFN [20–80 µM] for 48 h at 37 °C in normoxic or hypoxic conditions. At the end of the treatment, 10 µL of cells viability reagent was directly added to cells in culture medium and incubate for 1 h at 37 °C and 5% CO_2_. The absorbance was detected at 570 and 600 nm using a microplate reader (GENios, TECAN^®^, Mannedorf, Switzerland). Values were expressed as fold increases of the percentage of cell viability and reported as mean ± standard deviation (SD) of three independent experiments.

### 4.3. Determination of Apoptotic Cell Death

The apoptotic status was assessed by Muse^®^ Caspases 3/7 kit (Merck Millipore, Burlington, MA, USA). The kit allows for the determination of the count and the percentage of cells in different stages of apoptosis based on the activity of executioner caspases 3/7 in combination with a dead cell dye. Briefly, U87MG and U373MG cells were seeded in a 6 well plate at 2 × 10^5^ cells/well, incubated for 24 h and treated with SFN [20–80 µM] for 48 h at 37 °C in normoxic or hypoxic conditions. At the end of the treatment, the culture medium was discarded, and 50 µL of cell suspensions were incubated with 5 µL of Muse^®^ Caspase-3/7 working solution at 37 °C for 30 min. At the end of the incubation, 150 µL of 7-AAD working solution was added to the samples and run-on Muse^®^ Cell Analyzer (Merck Millipore). Values were expressed as percentage of live, early and late apoptotic cells and reported as mean ± SD of three independent experiments.

The cytoplasmatic histone-associated DNA fragmentation was determined using the kit Cell Death Detection ELISAplus (RocheDiagnostics GmbH, Mannheim, Germany), as previously described [66], according to the manufacturer’s instructions. Briefly, U87MG and U373MG cells were seeded in a 96 well plate at 1 × 10^4^ cells/well, incubated for 24 h and treated SFN [20–80 µM] for 48 h at 37 °C in normoxic or hypoxic conditions. At the end of the treatment, 200 µL of lysis buffer were added to each well for 30 min, and 20 µL of supernatant were then incubated in a streptavidin-coated 96-well plate with 80 µL of a mixture of two monoclonal antibodies–anti-histone (biotin-labeled) and anti-DNA (peroxidase-conjugated). Wells were washed with the incubation buffer, and 100 µL of peroxidase substrate solution (ABTS^®^) were added for 15 min. The Optical Density (OD) was measured at 405 nm using a microplate reader (GENios, TECAN^®^). Values were expressed as fold increases of DNA fragmentation versus corresponding controls and reported as mean ± SD of three independent experiments.

### 4.4. Analysis of Cell Cycle

The cell cycle distribution was assessed by Muse^®^ Cell Cycle kit (Merck Millipore), which allows the quantitative measurements of the percentage of cells in the G0/G1, S, and G2/M phases of cell cycle. Briefly, U87MG and U373MG cells were seeded in a 6 well plate at 2 × 10^5^ cells/well, incubated for 24 h and treated with SFN [20–80 µM] for 48 h at 37 °C in normoxic or hypoxic conditions. At the end of the treatment, the culture medium was discarded, and 200 µL of cell suspensions were centrifugated at 1500 rpm for 5 min before being washed with 1X phosphate buffered saline (PBS). Cells were then fixed adding 200 µL of ice cold 70% ethanol and kept at −20°C before being centrifugated at 1500 rpm for 5 min and washed again with 1X PBS. Two hundred µL of Muse^®^ Cell Cycle Reagent were added in each sample and incubated for 30 min at room temperature in the dark before being run on Muse^®^ Cell Analyzer (Merck Millipore). Values were expressed as the percentage of cells in the G0/G1, S, and G2/M phases and reported as mean ± SD of three independent experiments.

### 4.5. Determination of Cell Motility

Cell motility was evaluated using the wound healing assay. U87MG and U373MG cell lines were seeded in a culture-insert (ibidi^®^ culture-insert 2 well, ibidi GmbH, Martinsried, Germany) at a density of 2 × 10^4^ cells/well and incubated for 24 h. After allowing the cells to attach overnight, the culture-insert was removed, leaving a free gap on the dish of 500 µm. The cells were then treated with SFN 40 µM at 37 °C in normoxic or hypoxic conditions and photographed at 3, 6, 24, 48, 72 h with a Nikon TS 100F microscope (Nikon Instruments Spa, Campi Bisenzio, Florence, Italy) to capture the area of the gap at 10× magnification. The area of the gap was then calculated using ImageJ analysis. Values were expressed as the percentage of the wound area not covered and reported as mean ± SD of three independent experiments.

### 4.6. Determination of GSH Levels

GSH levels were determined by the GSH reductase-coupled 5,5′-dithiobis (2-nitrobenzoic acid) (DTNB) assay. U87MG and U373MG cells were seeded in a 6 well plate at 2 × 10^5^ cells/well, incubated for 24 h and treated with SFN [20–80 µM] for 48 h at 37 °C in normoxic or hypoxic conditions. Briefly, at the end of the treatment, cells were washed with cold PBS, collected in 1.5 mL of PBS and centrifuged for 10 min at 10,000 rpm at 4 °C. Pellets were then lysed with 500 μL of 0.1% Triton X-100, and centrifuged at 14,000 rpm for 15 min at 4 °C. Twenty-five µL of supernatant were collected into a 96-well plate and 25 μL of cold sulfosalicylic acid (5%) were added to each well. The plate was shaken for 2 min and 125 μL of the reaction buffer (containing the DTNB) were added. The plate was shaken for 15 s and the OD was measured at 405 nm using a microplate reader (GENios, TECAN^®^) for 5 min every 1 min. Values were calculated using a standard calibration curve and expressed as the mean of fold increases ± SD of three independent experiments.

### 4.7. Determination of Phospho-ERK1/2 and Cytochrome c

The phospho-ERK1/2 and cytochrome c were evaluated using the Western Blotting method [16,67]. U87MG and U373MG cells were seeded in 60 mm dishes at 2 × 10^6^ cells/dish, incubated for 24 h and treated with SFN [20–80 µM] for 48 h at 37 °C in hypoxic or normoxic conditions. At the end of incubation, cells were trypsinized and the cellular pellet was resuspended in complete lysis buffer containing leupeptin (2 µg/mL, Sigma-Aldrich), phenylmethylsulfonyl fluoride (PMSF, 100 µg/mL, Sigma-Aldrich) and a cocktail of protease/phosphatase inhibitors (100×). The protein concentration was determined using the Bradford method (Bio-Rad Laboratories Srl, Hercules, CA, USA). The protein lysates (30 µg per sample) were separated by Mini-PROTEAN TGX Stain-Free™ precast gels (4–15% SDS polyacrylamide gels, Bio-Rad Laboratories SrL) and electroblotted onto 0.45 µm nitrocellulose membranes. Membranes were incubated overnight at 4 °C with primary antibody recognizing phospho-p44/42 (p-ERK1/2) and cytochrome c (1:1000, Cell Signaling Technologies Inc, Danvers, MA, USA). Membranes were then washed with TRIS-buffered saline-T (TBS + 0.05% Tween20), and then incubated with horseradish peroxidase (POD) linked anti-rabbit secondary antibody (1:2000, GE Healthcare, Chicago, IL, USA). Immunoreactive bands were visualized by enhanced chemiluminescence (ECL, Bio-Rad Laboratories Srl). The same membranes were stripped and reprobed with total p44/42 (ERK1/2, 1:1000, Cell Signaling Technology Inc.) or anti β-actin (1:1000, Sigma-Aldrich). Data were normalized on the total protein bands and analyzed by densitometry, using the Quantity One software (Bio-Rad Laboratories Srl). Values are expressed as fold increases and reported as the mean ± SD of three independent experiments.

### 4.8. Statistical Analysis

Data are reported as the mean fold increases ± SD of at least three independent experiments. Statistical analysis was performed using two-way ANOVA with Bonferroni post hoc test, and differences were considered significant at *p* ˂ 0.05. Analysis was performed using PRISM 9 software (GraphPad Software, La Jolla, CA, USA).

## 5. Conclusions

Overall, our data indicated that SFN was effective in inducing S-phase cell-cycle arrest and inhibiting cell growth in GBM cells also under hypoxic condition and these effects may be attributed to its ability to increase oxidative status and phosphorylation of ERK1/2. SFN highlights a pro-apoptotic effect at 20 and 40 μM levels, which are within the physiological concentration achievable by eating a high amount of broccoli products or through supplementation [68]. The peculiarity of this compound is that it maintains its effect even in the hypoxic condition, which is a phenomenon common in a majority of malignant tumors, and it may represent a winning strategy towards resistance to the treatments available. Overall, our findings suggest that SFN causes apoptosis in GBM; however, further studies are needed to better explore the underlying mechanisms of its action, facilitating finding more therapeutic strategies for treating GBM. Several studies show how natural compounds could exert anti-GBM effects by upregulating apoptosis and autophagy, inducing cell cycle arrest, interfering with tumor metabolism, and inhibiting proliferation, neuroinflammation, chemoresistance, angiogenesis, and metastasis [56]. Although these beneficial effects are promising, the efficacy of natural substances in GBM is constrained by their bio-availability and BBB permeability, which, on the contrary, appear not to be a problem with SFN treatment. Our study may not only corroborate the chemopreventive activity of SFN, but also show new directions for the rational application of SFN in anticancer strategies against hypoxic tumors. A better understanding of the mechanisms by which SFN induces cell cycle arrest and apoptosis is crucial for its future development as a clinically valuable cancer preventive/therapeutic agent, and this information could contribute to the identification of mechanism-based biomarkers crucially involved in future clinical trials.

## Data Availability

The data presented in this study are available on request from the corresponding author.
